# *Enterococcus faecium* HDRsEf1 induces changes in gut microbiota and metabolites to maintain host health

**DOI:** 10.3389/fimmu.2026.1760841

**Published:** 2026-04-15

**Authors:** Shuaifei Feng, Cheng Tian, Jiaxuan Li, Wei Cheng, Shiyu Tao, Yuncai Xiao, Changchun Li, Hong Wei

**Affiliations:** 1 College of Animal Sciences and Technology, Huazhong Agricultural University, Wuhan, China; 2 National Key Laboratory of Agricultural Microbiology, Huazhong Agricultural University, Wuhan, China; 3 College of Veterinary Medicine, Huazhong Agricultural University, Wuhan, China; 4 Yu-Yue Pathology Scientific Research Center, Jinfeng Laboratory, Chongqing, China

**Keywords:** *Enterococcus faecium* HDRsEf1, gut metabolites, gut microbiota, inflammation, nursery pigs

## Abstract

Gut microbiota plays a pivotal role in host immunity and overall health. *Enterococcus faecium* HDRsEf1 (Ef1), a novel potential probiotic, has been reported to enhance immune defense against pathogenic invasion; however, its effects on inflammation and gut microbiota remain poorly understood. This study aimed to evaluate the impact of Ef1 on host health, gut microbiota, and fecal metabolite profiles using DSS-induced colitis in mice and nursery pig models. In mice, Ef1 effectively mitigated growth retardation and organ damage caused by DSS-induced inflammation. In nursery pigs, Ef1 administration reduced markers of inflammation and oxidative stress, including IL-1β, IL-6, TNF-α, and MDA, while increasing the anti-inflammatory cytokine IL-10 and the antioxidant enzyme SOD. Furthermore, Ef1 modulated gut microbiota composition, promoting beneficial genera such as *Ruminococcus* and *Acetobacter*, while suppressing potentially harmful genera like *Acinetobacter*. Metabolomic analysis revealed that Ef1 enriched anti-inflammatory metabolites in the gut. Collectively, these findings indicate that Ef1 can beneficially modulate gut microbiota and metabolite profiles, thereby enhancing host health.

## 1 Introduction

The gut microbiota is a complex and diverse ecosystem composed of bacteria, fungi, viruses, and other microorganisms. These microbes are capable of breaking down complex polysaccharides that the host cannot directly utilize, producing various metabolites that promote the digestion and absorption of nutrients from food (1). Studies have shown that gut microbiota plays an important role in host health by producing short-chain fatty acids (SCFAs) through the degradation of complex polysaccharides (2, 3), modulating host immunity (4–6), and maintaining the integrity of the intestinal barrier (7, 8). Furthermore, gut microbiota plays a critical role in regulating the host immune system. Through specific metabolites, modulation of immune cell activity, and influence on signaling pathways, gut microbiota not only maintains immune balance and defends against pathogen invasion but also prevents excessive immune responses (4, 5). These microbial activities form the basis for the regulation of host physiological and immune functions.

Probiotics possess various beneficial biological properties, including modulation of immune cell activity and expression of inflammatory factors, inhibition of pathogen growth, and prevention or alleviation of inflammation and tissue damage associated with various diseases (9). Probiotics also play a key role in maintaining gut health by secreting organic acids and antimicrobial substances that regulate the composition and function of the gut microbiota (9). *Enterococcus faecium* is a probiotic approved by the European Union and the US FDA for use in animal feed (10). It is characterized by its heat, acid, and bile salt tolerance, as well as its ability to adhere to the intestinal surface and inhibit the growth of pathogenic bacteria (11). The HDRsEf1 strain was isolated from the gut of Chinese Tongcheng pigs and has shown significant anti-inflammatory and epithelial-protective effects. Some studies have indicated that this strain can regulate TLR2/4-mediated signaling pathways, restore *ZO-1* expression, and inhibit TNF-α production (12, 13). Further research has shown that *E. faecium* HDRsEf1 (Ef1) can regulate pathogen-associated molecular patterns, enhance IL-12 expression, induce systemic Th1 immune responses, and improve antimicrobial defense against Salmonella Typhimurium (14). Additionally, previous studies have found that Ef1 can reshape the gut microbiota, alleviate inflammation, and support intestinal morphological development in weaned piglets (15). However, the role of Ef1 in inflammatory conditions and its effects on host metabolism remain unclear. This study evaluates the effects of Ef1 in a DSS-induced colitis mouse model to determine its specific roles in improving intestinal health and alleviating inflammation. The transition period from weaning to feeding in nursery pigs is a time when the intestinal barrier and microbiota are highly susceptible to infection, so this study also investigates the effects of feeding different concentrations (0.05% and 0.1%) of Ef1 powder to nursery pigs to determine its effective dose and impact. The results indicate that Ef1 effectively alleviated DSS-induced intestinal inflammation and growth retardation in mice. In nursery pigs, dietary supplementation with 0.1% Ef1 demonstrated the most pronounced efficacy, significantly improving average daily gain and modulating gut microbiota composition along with metabolite profiles. By employing multiple animal models, this study comprehensively evaluated the impact of Ef1 on host health. The findings provide critical insights and references for the development and application of Ef1 as a probiotic.

## 2 Materials and methods

### 2.1 Experimental animals

Twenty-six SPF male BALB/c mice (5 weeks old, 20.09 ± 1.41 g) were obtained from the Animal Experiment Center of Huazhong Agricultural University and housed under controlled conditions (25 ± 2 °C, 12 h light/dark cycle) with ad libitum access to food and water. Fifty-four male nursery pigs (Duroc × Landrace × Yorkshire, 35 days old, 8.66 ± 0.52 kg) were supplied by Guangxi Yangxiang Co., Ltd. and housed in ventilated pens with regular cleaning, ad libitum water, and a balanced diet provided three times daily. The feed composition is shown in **Supplementary Table S1**. Standard husbandry practices, including vaccination and biosecurity measures, were followed. At the conclusion of the study, mice were euthanized using a combination of chemical and physical methods to ensure minimal pain and distress. Initially, mice were deeply anesthetized via inhalation of 3% isoflurane until the loss of the pedal withdrawal reflex was confirmed. Subsequently, cervical dislocation was performed by trained personnel as a secondary physical method to ensure permanent cessation of vital functions. All procedures were approved by the Huazhong Agriculture University Institutional Animal Care and Use Committee and complied with the Guidelines for the Care and Use of Laboratory Animals (Protocol No. HZAUMO-2025-0525).

### 2.2 Experimental design

Twenty-six mice were randomly allocated into three groups: healthy control (CON, n = 6), DSS-induced colitis (CON-DSS, n = 10), and Ef1 intervention (Ef1-DSS, n = 10). Mice in the Ef1-DSS group received daily oral gavage of Ef1 suspension (100 μL, 1 × 10^8^ CFU), while CON and CON-DSS groups received PBS (100 μL). Gavage lasted 7 days, followed by administration of 3% DSS in drinking water for CON-DSS and Ef1-DSS groups from days 8 to 14; CON mice received regular water. Body weights were recorded daily. On day 15, blood was collected via orbital bleeding, and mice were euthanized for collection of spleen, colon, and rectum.

The 54 nursery pigs were randomly assigned to three groups: Control (basal diet, n = 18), low-dose Ef1 (0.05% HDRsEf1, n = 18), and high-dose Ef1 (0.1% HDRsEf1, n = 18), with six replicates per group and three pigs per replicate. The specification of HDRsEf1 bacterial powder is 2 × 10^9 CFU/g. The feeding trial lasted 28 days, during which body weights were recorded on days 0 and 28. Fecal and blood samples were collected on day 28 for analysis. The basal diet was formulated according to NRC (2012) recommendations.

### 2.3 Histopathological analysis

Distal colon tissues were fixed in 4% paraformaldehyde, embedded in paraffin, and sectioned into 3-μm slices using a Leica microtome. The sections were stained with hematoxylin and eosin (H&E) and examined under a Leica light microscope. Histopathological evaluation included assessment of inflammatory cell infiltration, epithelial injury, crypt damage, and overall lesion severity.

### 2.4 Enzyme-linked immunosorbent assay

Cytokine levels were measured in different matrices depending on the animal model to optimize detection sensitivity and physiological relevance. In the mouse model, colon tissue homogenates were utilized to capture site-specific inflammatory markers associated with mucosal damage. In the pig model, serum samples were employed to monitor the systemic inflammatory status, reflecting the overall physiological response to the dietary treatment. In mice, inflammatory cytokines (TNF-α, IL-1β, IL-6, and IL-10) were quantified in colon tissue. Colon samples were rinsed with PBS, minced, and homogenized on ice in pre-chilled PBS containing protease inhibitors at a 1:9 (w/v) tissue-to-buffer ratio. Homogenates were centrifuged at 10,000 × *g* for 10 min at 4 °C, and the resulting supernatants were collected for cytokine analysis using Bioswamp ELISA kits. In swine, serum concentrations of inflammatory cytokines (TNF-α, IL-1β, IL-6, and IL-10) and oxidative stress markers (SOD and MDA) were measured. Blood samples were allowed to clot at room temperature for 1 h and centrifuged at 3,000 × *g* for 10 min. The collected serum was analyzed using Bioswamp ELISA kits according to the manufacturer’s instructions.

### 2.5 RNA extraction and quantitative real-time PCR

Colon tissues were used to analyze the expression of tight junction–related genes (*ZO-1*, *Occludin*, and *Claudin-1*). Total RNA was extracted using TRIzol reagent (Thermo Fisher Scientific, MA, USA) following the manufacturer’s instructions. The extracted RNA was reverse-transcribed into cDNA using the Hifair^®^ III 1st Strand cDNA Synthesis SuperMix with gDNA digester (YEASEN, Shanghai, China). Gene expression was quantified by RT-qPCR using Hieff^®^ qPCR SYBR^®^ Green Master Mix (YEASEN, Shanghai, China). Relative expression levels were calculated using the 2^−ΔΔCT method, with GAPDH as the internal reference gene.

### 2.6 DNA extraction, 16S rRNA gene amplification, and sequencing

Genomic DNA was extracted from swine fecal samples using the OMEGA Soil DNA Kit (Omega Bio-Tek, GA, USA) according to the manufacturer’s instructions. DNA concentration and purity were assessed using a NanoDrop NC2000 spectrophotometer (Thermo Fisher Scientific, MA, USA) and agarose gel electrophoresis. The V3–V4 region of the bacterial 16S rRNA gene was amplified using primers 338F (5′-ACTCCTACGGGAGGCAGCA-3′) and 806R (5′-GGACTACHVGGGTWTCTAAT-3′). PCR products were purified with VAHTS™ DNA Clean Beads (Vazyme, Nanjing, China) and quantified using the Quant-iT PicoGreen dsDNA Assay Kit (Invitrogen, CA, USA). Sequencing was performed on the Illumina NovaSeq 6000 platform (500 cycles) at Shanghai Personal Biotechnology Co., Ltd.

### 2.7 Bioinformatics analysis

Microbiome bioinformatics analyses were performed using QIIME2 (v2019.4). Raw reads were demultiplexed with the demux plugin, and primer sequences were removed using cutadapt. Quality control, denoising, merging, and chimera removal were carried out using DADA2 (16). Alpha diversity (Chao1, observed species, Shannon, Simpson, Faith’s PD, Pielou’s evenness, and Good’s coverage) and beta diversity (Jaccard distance) were calculated using the diversity plugin and visualized in R with ggplot2. Differences in community structure were assessed by PERMANOVA (adonis function, vegan, 999 permutations). Taxonomic classification of ASVs was performed using the classify-sklearn naïve Bayes classifier in the feature-classifier plugin against the Greengenes 13_8 reference database (99% similarity). Functional profiling was inferred using PICRUSt2. KEGG pathway enrichment significance was determined using Fisher’s exact test, and p-values were adjusted for multiple testing using the Benjamini-Hochberg (FDR) method. Key discriminatory microbial taxa were identified using LEfSe and random forest analysis.

### 2.8 Untargeted metabolomics analysis

Swine fecal samples were thawed at 4 °C and extracted with 1 mL of precooled methanol/acetonitrile/water (2:2:1, v/v), followed by vortexing and sonication on ice for 30 min. Samples were incubated at −20 °C for 10 min to precipitate proteins and centrifuged (14,000 × *g*, 20 min, 4 °C). Supernatants were dried under vacuum and stored at −80 °C. Prior to LC-MS/MS analysis, dried samples were reconstituted in 100 μL acetonitrile/water (1:1, v/v), vortexed, and centrifuged (14,000 × *g*, 15 min, 4 °C).

Metabolite separation was performed on an UHPLC system (Agilent 1290 Infinity LC) using HILIC and RPLC methods with mobile phases buffer A (water with 25 mM ammonium acetate and ammonium hydroxide) and buffer B (acetonitrile). System stability was monitored using QC samples. Metabolites were analyzed in both positive and negative ESI modes on an AB SCIEX TripleTOF 6600. Raw MS data were converted to MzXML format and processed with XCMS for feature detection, alignment, and retention time correction. Metabolites were identified by matching accurate mass (<25 ppm) and MS/MS spectra to an in-house database. Features present in ≥50% of samples in any group were retained.

Normalized datasets for positive and negative ion modes were imported into R for statistical analyses. PCA and OPLS-DA (*ropls* package) were used to evaluate metabolic changes. Differential metabolites were visualized with volcano plots and heatmaps, using VIP > 1.0 and *p* < 0.05 (t-test) as thresholds. Spearman correlations among differential metabolites, microbial markers, inflammatory cytokines, and oxidative stress indicators were calculated using the *corrplot* and *pheatmap* packages in R.

### 2.9 Statistical analysis

For nursery pigs, each replicate was considered an experimental unit, and mean values per replicate were calculated. Data are presented as mean ± SEM. Normally distributed data were analyzed using independent t-tests for two-group comparisons or one-way ANOVA followed by Tukey’s *post hoc* test. Non-normally distributed data were analyzed using the Kruskal-Wallis test, with pairwise comparisons adjusted by the Benjamini-Hochberg (BH) method. Statistical significance was defined as *p* < 0.05. Spearman correlation analysis was performed to evaluate the associations among differential microbial taxa, metabolites, and host phenotypes. Multiple testing for all correlation analyses was corrected using the Benjamini-Hochberg (BH) method to control the false discovery rate (FDR), and significant associations were identified based on the adjusted *p*-values (*q* < 0.05). All analyses and visualizations were performed in R (version 4.3.1).

## 3 Results

### 3.1 HDRsEf1 alleviates growth retardation and organ lesions induced by DSS in mice

**Figure 1:**
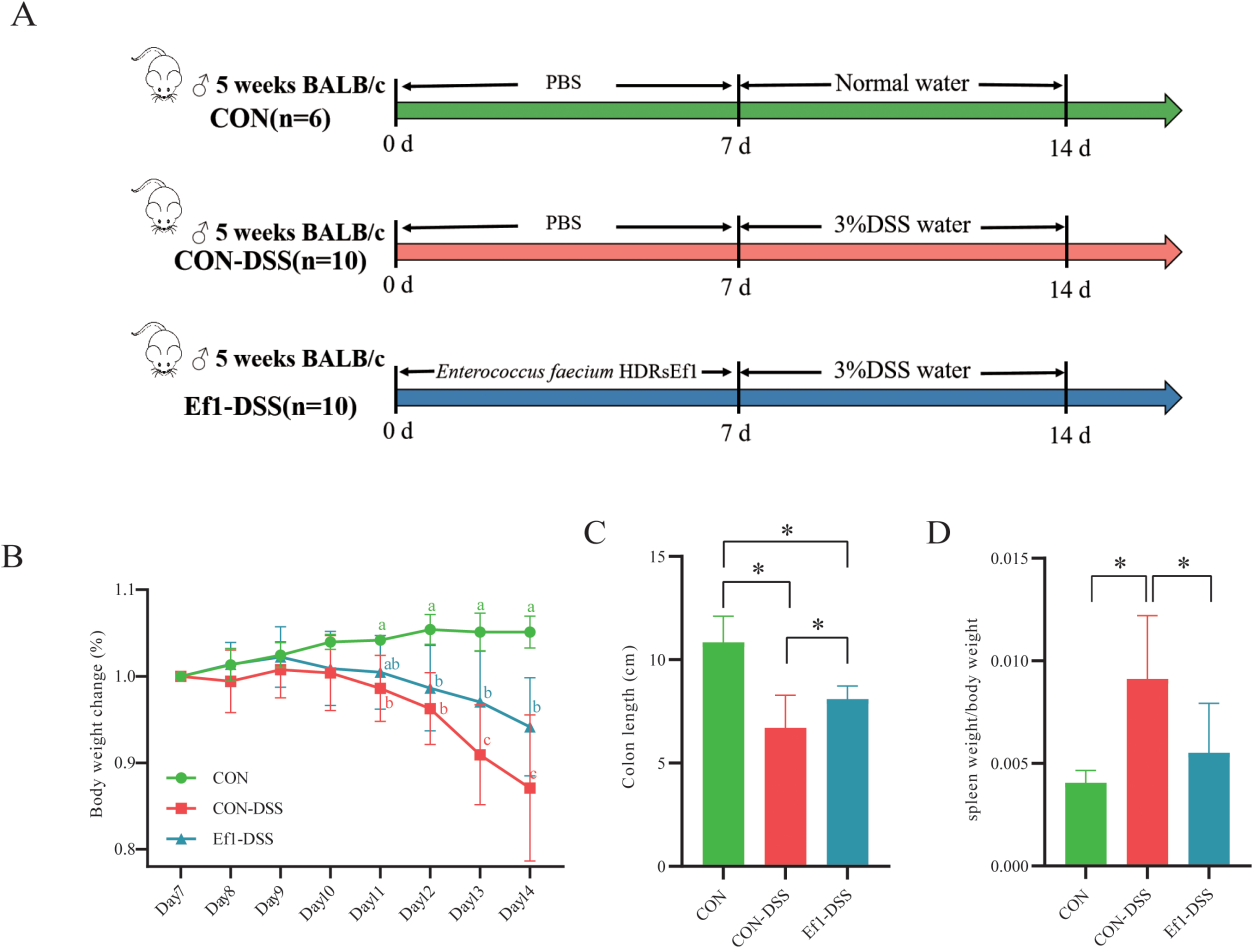
HDRsEf1 alleviates DSS-induced growth retardation and organ pathology in mice. **(A)** Experimental design of the DSS-induced colitis mouse model and Ef1 intervention. **(B)** Body weight changes during the experimental period. Significant differences are indicated by different letters. **(C)** Colon length measurements. **(D)** Spleen index of mice. Statistical significance was determined by one-way ANOVA followed by Tukey’s *post hoc* test. *P* < 0.05 was considered statistically significant. *: *p* < 0.05.

To assess the effects of HDRsEf1 (Ef1) on intestinal inflammation, mice were divided into three groups, and colitis was induced with DSS (**Figure 1A**). Weight loss is a key indicator of DSS-induced ulcerative colitis (UC); therefore, body weights were monitored daily from day 7, when DSS administration began. Between days 7 and 10, no significant differences in weight gain were observed among the groups. From day 11 onward, the CON group exhibited significantly higher weight gain compared to the CON-DSS groups (*p* < 0.05), confirming successful induction of growth retardation. By day 13, the Ef1-DSS group showed significantly higher body weight than the CON-DSS group (*p* < 0.05), indicating that Ef1 partially mitigates DSS-induced growth impairment (**Figure 1B**). Colon shortening and spleen enlargement are hallmark features of DSS-induced UC. Dissection measurements revealed that the CON group had significantly longer colons and lighter spleens compared to the CON-DSS (*p* < 0.05) (**Figures 1C, D**), confirming DSS-induced organ lesions. Importantly, the Ef1-DSS group exhibited significantly longer colons and reduced spleen weights relative to the CON-DSS group (*p* < 0.05), suggesting that Ef1 confers protective effects against DSS-induced organ damage. Overall, these results demonstrate that Ef1 can alleviate DSS-induced growth retardation and organ lesions, providing partial resistance to inflammation in this UC mouse model.

### 3.2 HDRsEf1 alleviates DSS-induced intestinal inflammation in mice

**Figure 2:**
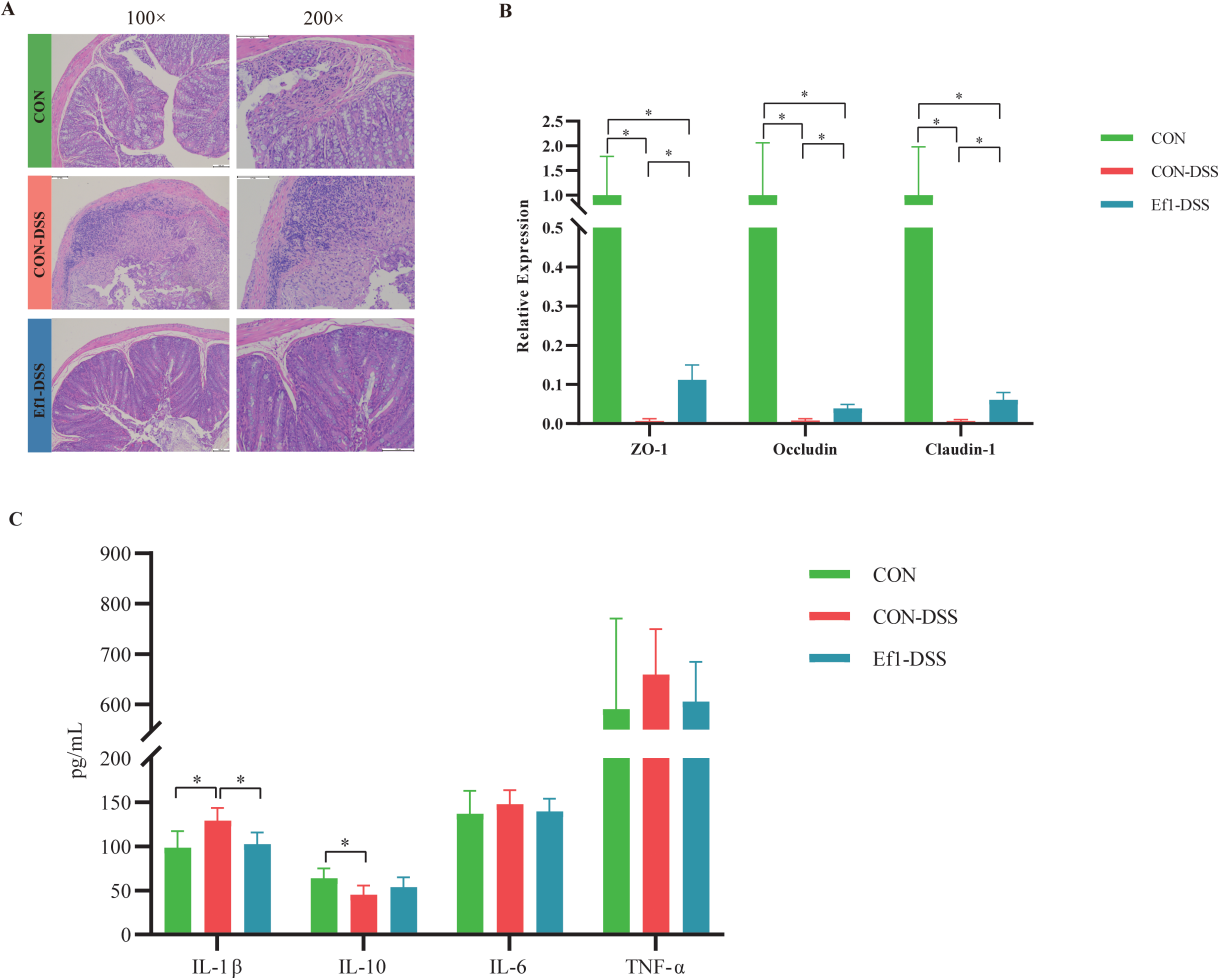
Ef1 supplementation attenuates DSS-induced colonic histopathological damage in mice. **(A)** Representative H&E-stained images of colon sections from each group. **(B)** Relative abundance of *ZO-1*, *Occludin*, and *Claudin-1* across experimental groups. **(C)** Measurement results of inflammatory markers (IL-1β, IL-10, IL-6, TNF-α) across experimental groups. Data are shown as mean ± SEM. Statistical analysis was performed using T-test. *p < 0.05.

Histological analysis of mouse colon tissue using H&E staining revealed intact tissue architecture in the CON group, with well-defined mucosal layers, villi, and crypts. Goblet cells were abundant, and no signs of inflammation or tissue damage were observed. In contrast, the CON-DSS group exhibited severe histopathological changes, including disruption of the mucosal layer, incomplete crypts, reduced goblet cells, and extensive infiltration of inflammatory cells, indicating a pronounced inflammatory response. The Ef1-DSS group showed partial restoration of colon structure, with increased goblet cells and reduced inflammatory cell infiltration, demonstrating notable improvement relative to the CON-DSS group (**Figure 2A**). To evaluate intestinal barrier integrity, the expression of tight junction genes *ZO-1*, *Occludin*, and *Claudin-1* was measured by RT-qPCR. Compared with the CON group, the CON-DSS and Ef1-DSS groups exhibited significantly reduced expression of all three genes (*p* < 0.05). Importantly, Ef1-DSS mice displayed significantly higher expression levels than the CON-DSS group (*p* < 0.05), suggesting that Ef1 partially mitigates DSS-induced disruption of the intestinal barrier (**Figure 2B**). Inflammatory cytokine levels in colon tissue were assessed by ELISA. IL-1β was significantly elevated in the CON-DSS group compared to both the CON and Ef1-DSS groups (*p* < 0.05), with no significant difference between CON and Ef1-DSS. IL-10 levels were lower in the CON-DSS group than in the Ef1-DSS group, and significantly lower than in the CON group. For IL-6 and TNF-α, levels in the Ef1-DSS group were lower than in the CON-DSS group (*p* > 0.05) and comparable to the CON group (**Figure 2C**). These findings indicate that Ef1 exerts anti-inflammatory effects, attenuating DSS-induced intestinal inflammation. In summary, Ef1 supplementation in mice protects against DSS-induced disruption of the intestinal barrier and inflammation, supporting its role in maintaining intestinal health.

### 3.3 Effect of Ef1 supplementation on average daily gain in nursery pigs

**Figure 3:**
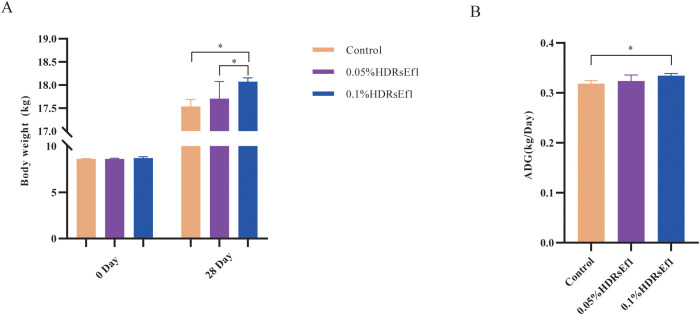
Effect of Ef1 supplementation on average daily gain (ADG) in nursery pigs. **(A)**Comparison of body weight in experimental pigs between Day 0 and Day 28; **(B)** Comparison of average daily gain (ADG) in experimental pigs. Statistical significance was determined by one-way ANOVA followed by Tukey’s *post hoc* test. *p* < 0.05 was considered statistically significant. *p < 0.05.

Following the protective effects observed in mice, we evaluated the impact of Ef1 in a larger, more robust model using nursery pigs, whose intestinal tissues and microbiota are particularly susceptible during the transition from maternal milk to artificial feed. Nursery pigs were divided into three groups: a control group fed a standard diet, and two experimental groups receiving 0.05% or 0.1% HDRsEf1 supplementation (based on feed proportion) for 28 days. To minimize stress, body weights were recorded only at the start and end of the feeding period, and average daily gain (ADG) was calculated. No significant differences in initial body weight were observed among the groups (*p* > 0.05) (**Figure 3A**). After 28 days, Nursery pigs in the 0.1% HDRsEf1 group exhibited significantly higher body weight compared to both the control and 0.05% HDRsEf1 groups (*p* < 0.05). Correspondingly, ADG was highest in the 0.1% HDRsEf1 group, showing a significant increase relative to the control group (*p* < 0.05) and a non-significant trend toward higher gain than the 0.05% HDRsEf1 group (*p* > 0.05). These results indicate that Ef1 supplementation promotes growth in nursery pigs, with the 0.1% inclusion level demonstrating superior efficacy.

### 3.4 Effects of Ef1 supplementation on serum inflammatory and oxidative stress markers in nursery pigs

**Figure 4:**
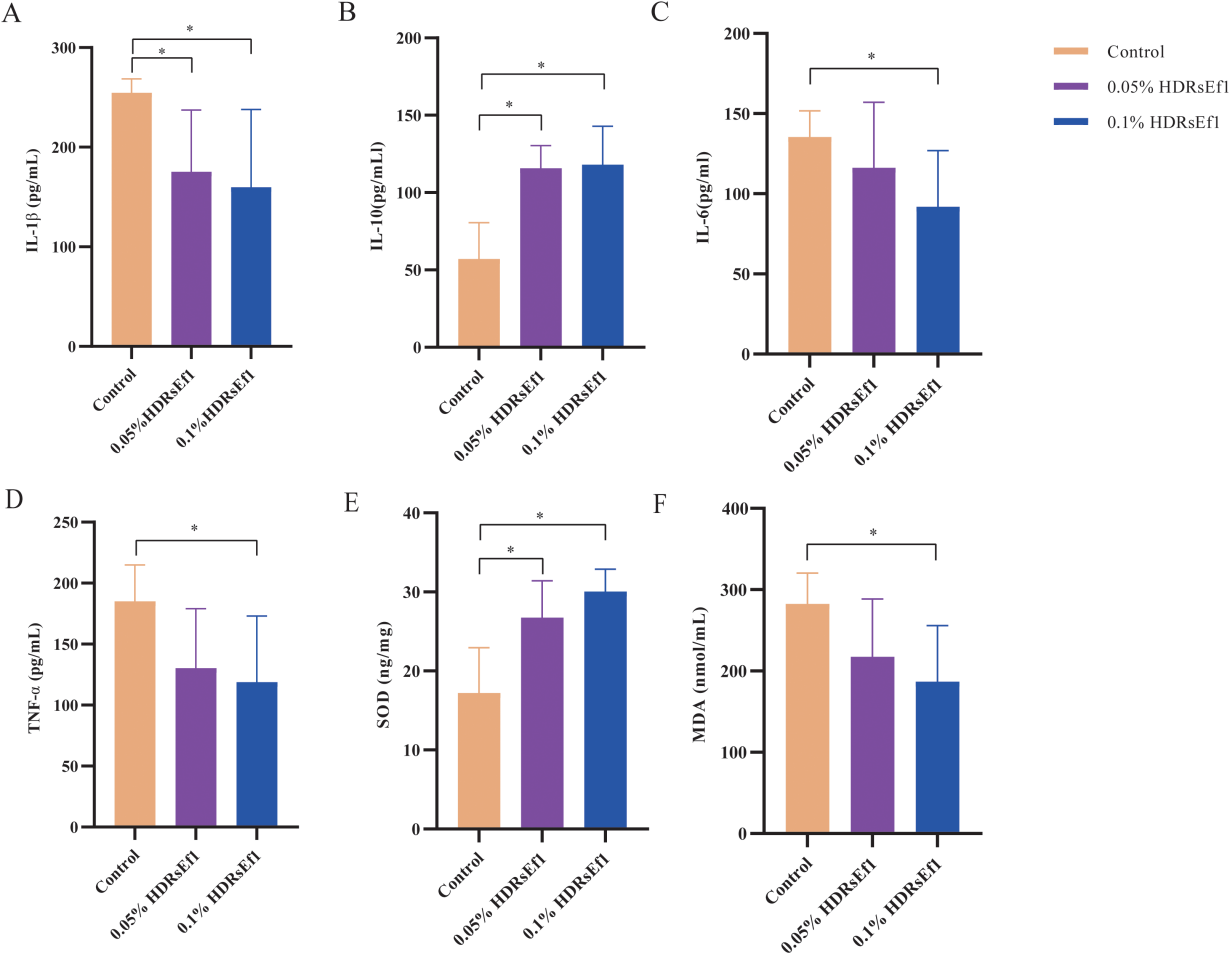
Effects of Ef1 supplementation on serum inflammatory and oxidative stress markers in nursery pigs. **(A–F)** Serum levels of IL-1β, IL-6, TNF-α, IL-10, SOD, and MDA in the Control, 0.05% HDRsEf1, and 0.1% HDRsEf1 groups. Statistical significance was determined by one-way ANOVA followed by Tukey’s *post hoc* test. *p* < 0.05 was considered statistically significant. *: *p* < 0.05.

To evaluate the impact of Ef1 on inflammation and oxidative stress, serum levels of IL-1β, IL-6, TNF-α, IL-10, SOD, and MDA were measured in nursery pigs. As shown in **Figures 4A–F**, the 0.1% HDRsEf1 group exhibited significantly lower levels of IL-1β, IL-6, TNF-α, and MDA compared to the control group (*p* < 0.05), and slightly lower levels than the 0.05% HDRsEf1 group (*p* > 0.05). Conversely, IL-10 and SOD levels were significantly elevated in the 0.1% HDRsEf1 group relative to both the control and 0.05% HDRsEf1 groups (*p* < 0.05). In the 0.05% HDRsEf1 group, IL-1β was significantly reduced (*p* < 0.05), and IL-10 and SOD were significantly increased (*p* < 0.05) compared with the control, whereas the other markers showed no significant differences. These findings indicate that Ef1 supplementation effectively mitigates inflammation and oxidative stress in nursery pigs, with the 0.1% inclusion level exerting the most pronounced effect.

### 3.5 Ef1 supplementation enriches beneficial bacteria in the gut microbiota of nursery pigs

**Figure 5:**
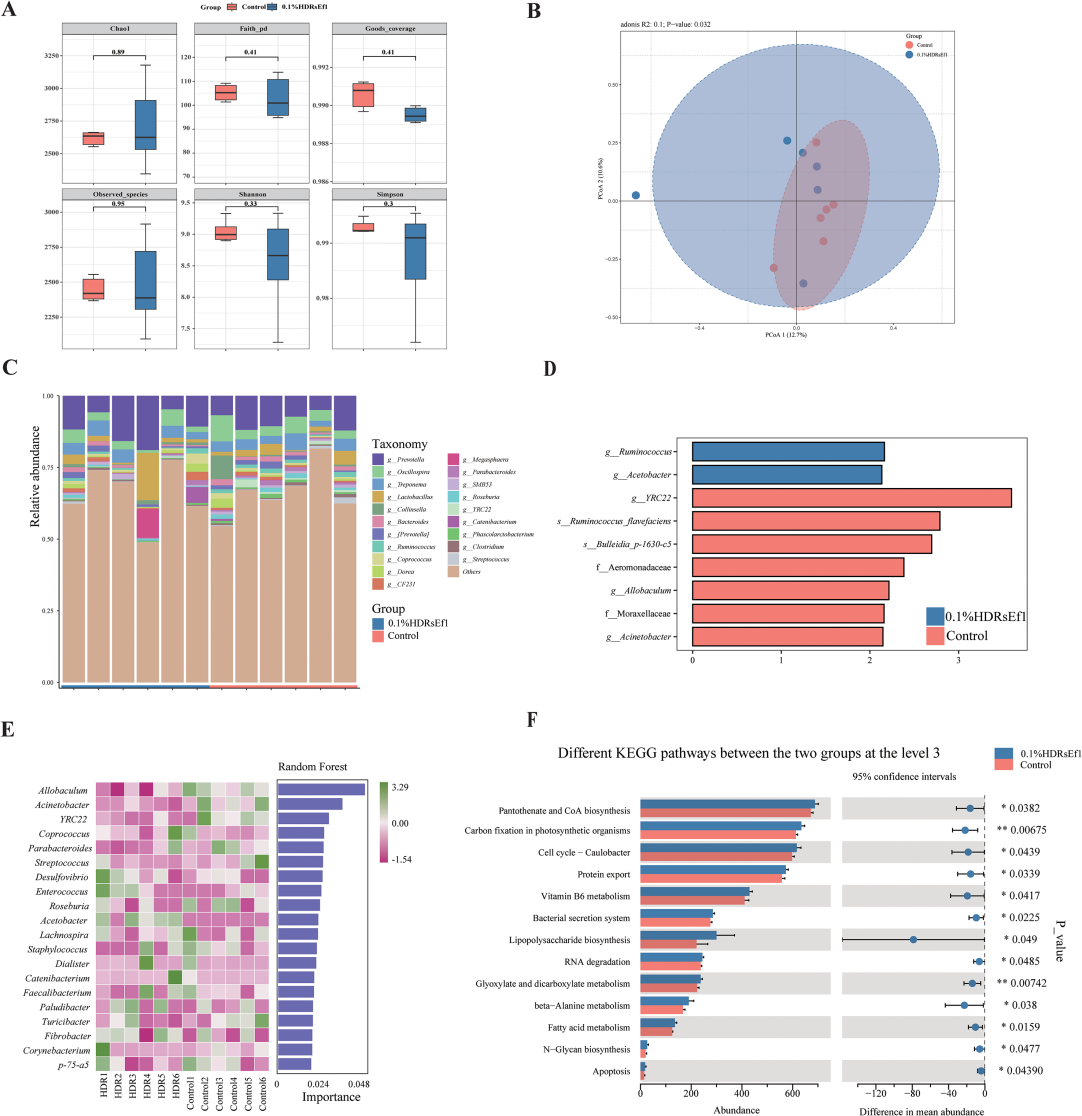
Effects of Ef1 supplementation on gut microbiota composition and function in nursery pigs **(A)** Alpha diversity indices (Chao1, Shannon, Simpson, Observed species) with error bars indicating SEM; **(B)** Principal coordinate analysis (PCoA) based on Jaccard distance; ADONIS test revealed significant separation between groups (*p* < 0.05). **(C)** Stacked bar chart of the top 10 genera by relative abundance. **(D)** Differentially abundant taxa identified by LEfSe analysis (LDA score > 2.0), with branch colors indicating the enriched group. **(E)** Key discriminative genera selected by random forest analysis. **(F)** Significantly altered KEGG Level 3 pathways (T-test, *p* < 0.05).

Based on previous findings, the 0.1% Ef1 supplement demonstrated beneficial effects on growth and health in nursery pigs. Therefore, fecal samples collected on day 28 from the 0.1% HDRsEf1 group and the control group were subjected to 16S rRNA amplicon sequencing. Rarefaction curve analysis indicated that sequencing depth was sufficient to capture the majority of microbial species (**Supplementary Figure S1A**). Alpha diversity indices showed no significant differences between groups (*p* > 0.05; **Figure 5A**), indicating comparable species richness and diversity. Principal Coordinate Analysis (PCoA) based on Jaccard distance, coupled with ADONIS testing, revealed a significant difference in microbial community composition between the two groups (*p* < 0.05; **Figure 5B**). At the phylum level, Firmicutes and Bacteroidetes dominated, accounting for over 90% of total sequences (**Supplementary Figure S1B**). At the genus level, the 0.1% HDRsEf1 group was enriched in *Prevotella*, *Lactobacillus*, and *Treponema*, whereas the control group was dominated by *Prevotella*, *Oscillospira*, and *Treponema* (**Figure 5C**). LEfSe analysis identified 2 species, 5 genera, and 2 families with significant differences between groups. Notably, the 0.1% HDRsEf1 group showed enrichment of *Ruminococcus* and *Acetobacter*, while genera such as *YRC22*, *Allobaculum*, *Acinetobacter*, and species *Ruminococcus flavefaciens* and *Bulleidia p_1630_c5* were significantly reduced (**Figure 5D**). Random Forest analysis further highlighted *Allobaculum*, *Acinetobacter*, and *YRC22* as key discriminative genera distinguishing the 0.1% HDRsEf1 group from the control (**Figure 5E**). Functional prediction using PICRUSt2 based on the KEGG database identified 6,773 KEGG Orthologs (KOs). PCoA of KO profiles showed clear separation between groups (**Supplementary Figure S1C**). At KEGG Level 1, microbial functions were predominantly associated with metabolism. At Level 2, main pathways included carbohydrate metabolism, amino acid metabolism, and metabolism of cofactors and vitamins (**Supplementary Figure S1D**). Notably, the 0.1% HDRsEf1 group showed higher enrichment in pathways related to glycan biosynthesis and metabolism, as well as cellular growth and death (**Supplementary Figure S1E**). At KEGG Level 3 (**Figure 5F**), functions such as pantothenate and CoA biosynthesis, carbon fixation in photosynthetic organisms, and the cell cycle (Caulobacter) were significantly predicted to be enriched in the 0.1% HDRsEf1 group. These results indicate that Ef1 supplementation is associated with changes in gut microbiota composition and function in nursery pigs, promoting the enrichment of beneficial bacteria such as *Lactobacillus*, *Ruminococcus*, and *Acetobacter*, which are associated with short-chain fatty acid production, and is associated with the predicted enhancement of metabolic pathways related to glycan synthesis and cellular growth.

### 3.6 Ef1 supplementation altered the fecal microbial metabolome in nursery pigs

**Figure 6:**
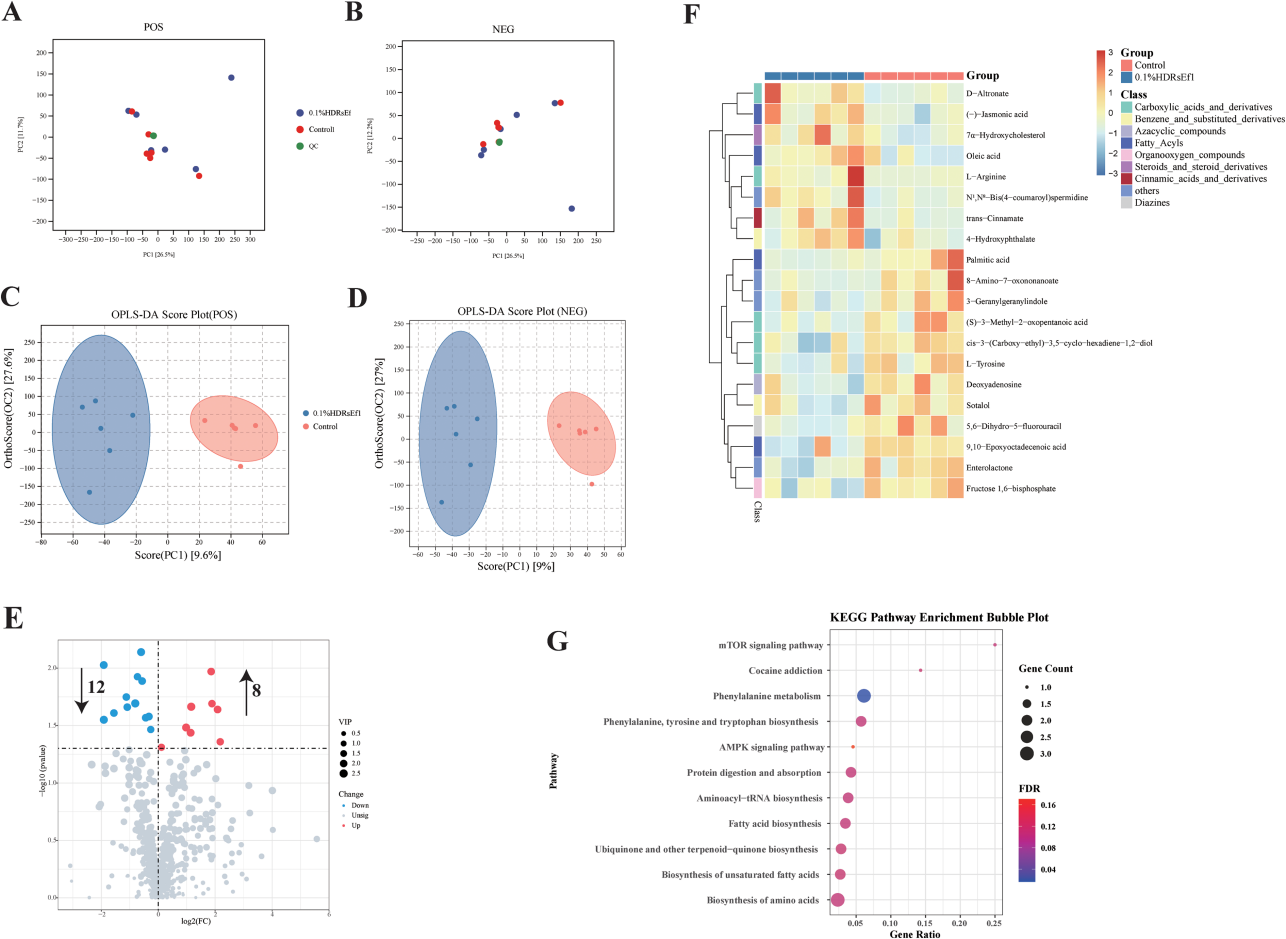
Ef1 supplementation alters the fecal metabolomic profile in nursery pigs **(A, B)** Principal component analysis (PCA) of QC samples and experimental samples in positive **(A)** and negative **(B)**. **(C, D)** Orthogonal partial least squares-discriminant analysis (OPLS-DA) score plots showing clear separation between the Control and 0.1% HDRsEf1 groups in positive **(C)** and negative **(D)** ion modes. **(E)** Volcano plot of differential metabolites in positive ion mode. Red dots represent enriched metabolites and blue dots represent depleted metabolites in the 0.1% HDRsEf1 group compared to the Control group (VIP > 1.0, *p* < 0.05). **(F)** Hierarchical clustering heatmap of significantly altered metabolites (VIP > 1.0, *p* < 0.05) across the two groups. Rows represent metabolites and columns represent individual samples; color intensity indicates relative abundance after Z-score normalization. **(G)** KEGG pathway enrichment analysis of differential metabolites. The vertical axis shows enriched metabolic pathways, and the horizontal axis represents the enrichment factor. Dot size indicates the number of metabolites mapped to each pathway, and dot color reflects the adjusted *p*-value (Fisher’s exact test with Bonferroni correction).

Fecal microbial metabolites play essential roles in regulating host physiology. To investigate the effects of Ef1 supplementation on metabolic profiles in nursery pigs, untargeted fecal metabolomics was conducted using LC-MS/MS. Quality control (QC) samples clustered tightly and separately from experimental samples, demonstrating high data quality and analytical stability (**Figures 6A, B**). Orthogonal Partial Least Squares Discriminant Analysis (OPLS-DA) further revealed a distinct separation between the two groups in both positive and negative ion modes (**Figures 6C, D**). The reliability of the OPLS-DA model was confirmed through permutation testing (**Supplementary Figures S2A, B**). Metabolite identification was performed using public and in-house metabolomics databases (17, 18), following the annotation criteria proposed by the Metabolomics Society in 2017 (19). A total of 398 metabolites were identified in positive ion mode and 197 in negative ion mode. Classification analysis revealed that the top 10 metabolite categories included carboxylic acids and derivatives, fatty acyls, benzenes and substituted derivatives (**Supplementary Figure S2C**). Based on Variable Importance in Projection (VIP > 1) and statistical significance (*p* < 0.05), 20 differential metabolites were selected as potential metabolic biomarkers. Several metabolites—such as D-arabinonic, L-Arginine, (-)-Jasmonic acid, 7α-Hydroxycholesterol, oleic acid, N¹, N^8^-bis(4-coumaroyl) spermidine, trans-Cinnamic, and H-hydroxyphthalic—were significantly enriched in the 0.1% HDRsEf1 group. In contrast, 12 metabolites, including palmitic acid, 8-amino-7-oxononanoic acid, and 3-terpenylindole, were significantly reduced in the 0.1% HDRsEf1 group (**Figures 6E, F**). To further understand the biological significance of these changes, KEGG pathway enrichment analysis was conducted using Fisher’s exact test with Bonferroni correction. Eighteen pathways were significantly enriched (*p* < 0.05), although two pathways remained significant after FDR correction (**Supplementary Table S2**), including phenylalanine metabolism, phenylalanine, tyrosine and tryptophan biosynthesis, aminoacyl-tRNA biosynthesis, protein digestion and absorption, fatty acid biosynthesis, and mTOR signaling, among others (**Figure 6G**). Collectively, these findings demonstrate that Ef1 supplementation induces profound alterations in the fecal metabolome of nursery pigs, modulating multiple bioactive metabolites and metabolic pathways related to amino acid synthesis, lipid metabolism, and cellular regulatory processes.

### 3.7 Significant correlations among intestinal microbiota, fecal metabolites, inflammatory markers, and oxidative stress indicators in nursery pigs

**Figure 7:**
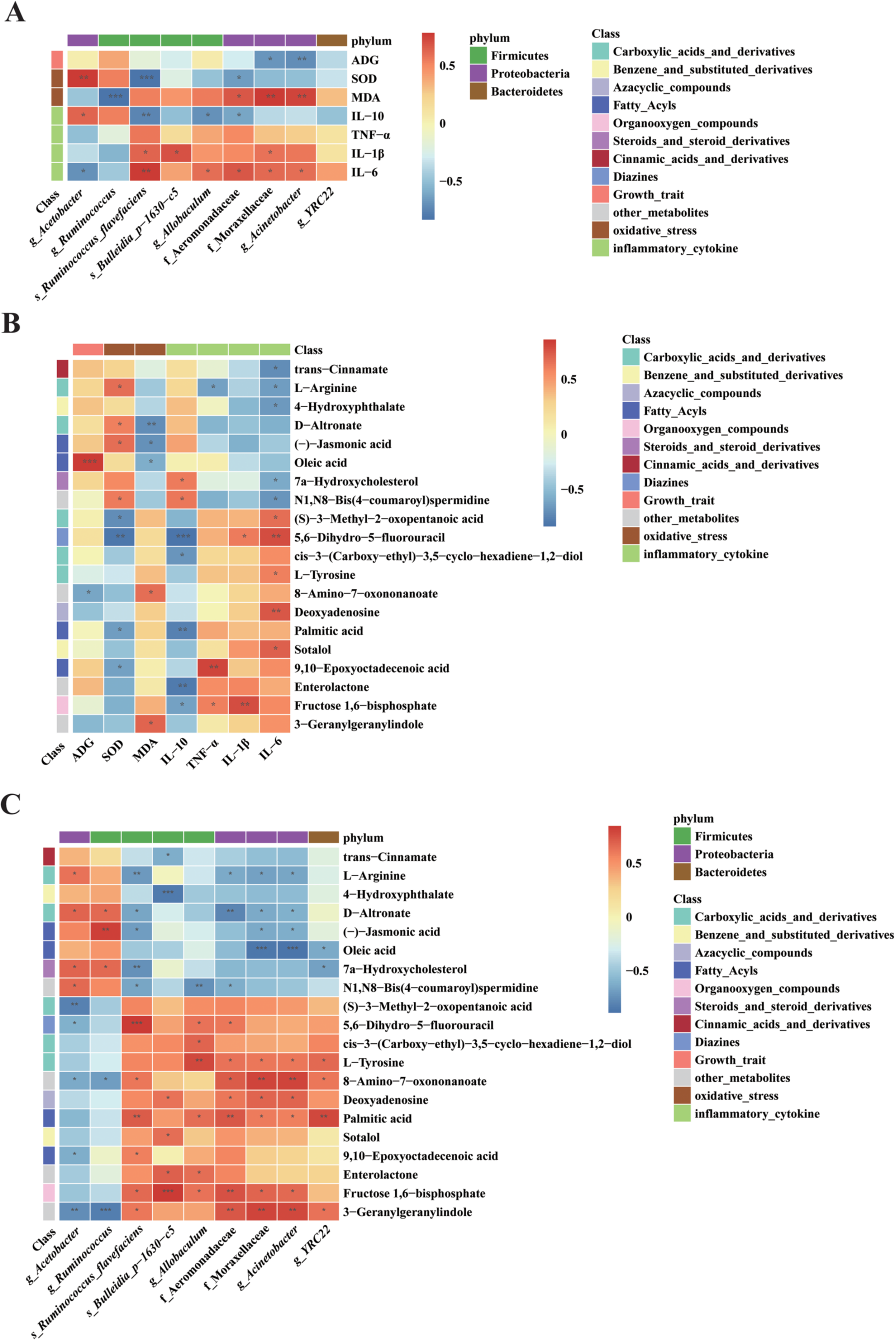
Integrated correlation analysis among gut microbiota, differential metabolites, and host-phenotypes. **(A)** Correlation between differential microbiota and host phenotypes. Heatmap showing Spearman correlation coefficients between key microbial taxa and growth performance (ADG), inflammatory cytokines (IL-6, IL-1β, IL-10, TNF-α), and oxidative stress markers (SOD). **(B)** Correlation between differential metabolites and host phenotypes. Heatmap illustrating the associations between significantly altered metabolites and the aforementioned host physiological indicators. **(C)** Correlation between differential microbiota and metabolites. Heatmap reflecting the intrinsic associations between the microbial community and the metabolic profile. In all panels, the color scale represents the Spearman correlation coefficient (r), where red indicates a positive correlation and blue indicates a negative correlation. Asterisks denote statistical significance after Benjamini-Hochberg FDR correction: *q < 0.05, **q < 0.01.

To comprehensively evaluate the integrated effects of Ef1 supplementation on various physiological factors in nursery pigs, Spearman correlation analyses were performed among differential microbiota, growth performance (ADG), inflammatory and oxidative stress markers, and differential metabolites. As shown in **Figure 7A** (Microbiota vs. Phenotypes), *Ruminococcus flavefaciens* was significantly and positively associated with pro-inflammatory cytokines (IL-6 and IL-1β), while showing negative correlations with the anti-inflammatory cytokine IL-10 and the antioxidant enzyme SOD. Similarly, *Moraxellaceae* and *Acinetobacter* were positively correlated with pro-inflammatory factors and negatively correlated with ADG. Consistent with these microbial trends, **Figure 7B** (Metabolites vs. Phenotypes) revealed that 5,6-Dihydro-5-fluorouracil was positively correlated with IL-6 and IL-1β, and negatively correlated with IL-10 and SOD. Furthermore, Fructose 1,6-bisphosphate exhibited significant positive correlations with IL-1β and TNF-α, and a negative correlation with IL-10. Integrated analysis in **Figure 7C** (Microbiota vs. Metabolites) demonstrated that these metabolites were significantly correlated with the aforementioned microbial taxa (e.g., *Ruminococcus flavefaciens* and *Acinetobacter*), which were enriched in the Control group. These associations suggest that these specific taxa and their related metabolites are correlated with poorer health and growth outcomes in nursery pigs. In contrast, *Acetobacter*—which was significantly enriched in the Ef1-supplemented group—exhibited a distinct association pattern. It was negatively correlated with IL-6 and positively correlated with IL-10 and SOD (**Figure 7A**). Its associated metabolite, N1,N8-Bis(4-coumaroyl) spermidine, showed a consistent correlation pattern with these host phenotypes (**Figures 7B, C**), suggesting a potential link between *Acetobacter*, its metabolic profile, and enhanced anti-inflammatory and antioxidant status.

In summary, these results indicate that Ef1 supplementation is associated with a shift in the gut microbial structure, characterized by an enrichment of beneficial taxa (such as *Acetobacter*) and a reduction in potentially harmful taxa found in nursery pigs. The concurrent alterations in microbial composition and metabolite profiles are closely linked to improved inflammatory and oxidative stress status, which may contribute to the observed enhancement in the growth performance of nursery pigs.

## 4 Discussion

The probiotic *Enterococcus faecium* HDRsEf1, which possesses notable anti-inflammatory properties, has been demonstrated in prior studies to promote a systemic Th1 immune response and enhance host resistance against *Salmonella* Typhimurium infection (14, 15). Based on preliminary experiments, a 3% DSS concentration was selected for this study to optimally treat mice and evaluate the anti-inflammatory and growth-promoting capacity of Ef1. Weight loss, colon shortening, and splenomegaly are hallmark pathological features of DSS-induced colitis in mice (20, 21). The results demonstrated significant weight loss, a marked reduction in colon length, and a significant increase in the spleen index in DSS-treated mice compared with the control group, confirming the successful establishment of the DSS-induced colitis model. Furthermore, Ef1 supplementation effectively mitigated these DSS-induced pathological responses. Our findings revealed that DSS significantly downregulated the expression of *Occludin*, *Claudin-1*, and *ZO-1* in the colon. Notably, Ef1 treatment alleviated this DSS-induced damage to the intestinal barrier. Inflammation is a key pathological mechanism in ulcerative colitis (22). Under normal physiological conditions, the intestinal immune system maintains tolerance to preserve barrier integrity. However, in UC, this balance is disrupted, leading to excessive release of pro-inflammatory cytokines that exacerbate both local and systemic inflammation. IL-1β, IL-6, and TNF-α serve as important indicators reflecting the severity of inflammation (23), whereas IL-10, an anti-inflammatory cytokine, suppresses the release of pro-inflammatory factors and mitigates inflammation (24). In this study, Ef1 significantly reduced DSS-induced IL-1β expression and increased IL-10 levels, highlighting its protective role in modulating intestinal inflammation. In summary, Ef1 alleviated the growth impairment and organ pathology induced by DSS in mice.

This study not only validated the experimental effects in a small animal model (mice) but also conducted a large-scale trial using a large animal model (nursery pigs), which is evolutionarily closer to humans. Evidence suggests that DSS-induced colitis parallels neonatal piglet intestinal inflammation in key aspects, notably epithelial barrier impairment, pro-inflammatory signaling, and microbial imbalance. These models overlap significantly in their pathological and metabolic signatures. Given that the porcine weaning immune system mirrors the “immaturity” of human infants, it provides a robust large-animal platform for testing anti-inflammatory therapies. Notably, DSS-induced lesions closely resemble those of Necrotizing Enterocolitis (NEC), a condition typically studied using piglet models. During acute inflammation, both models show depleted colonic SCFAs and lactate. The observation that metabolite supplementation alleviates symptoms across both rodent and porcine models underscores a conserved metabolic regulatory pathway (25–28). Unlike previous studies, the experimental design took clinical practicality into consideration by incorporating two concentration gradients to examine the dose-response relationship. The results ultimately demonstrated that a 0.1% bacterial powder-to-feed ratio yielded more pronounced effects. Due to considerations related to production efficiency in farming settings, the experiment did not involve dissection of the experimental pigs. Instead, the focus was on evaluating the impact of the bacterial strain based on phenotypic data, including average daily gain (ADG) and serum markers of inflammation and oxidative stress.

Analysis of ADG data revealed that Ef1 significantly promoted daily weight gain in nursery pigs. However, to avoid stress in the nursery pigs, body weight was not recorded multiple times during the trial. Furthermore, constraints related to equipment and practical operations prevented the collection of feed intake and feed conversion ratio data. These factors warrant further investigation. Serum levels of inflammatory markers and oxidative stress indicators—including TNF-α, IL-6, IL-1β, IL-10, SOD, and MDA—were also measured. Superoxide dismutase (SOD), a key antioxidant enzyme, mitigates oxidative damage by neutralizing superoxide radicals, while malondialdehyde (MDA), a terminal product of lipid peroxidation, is typically maintained at low levels in healthy individuals (29). The results indicated that Ef1 effectively reduced both inflammatory and oxidative stress levels.

Furthermore, we examined the intestinal microbiota and metabolome data of the experimental pigs at the end of the trial to investigate the impact of Ef1 on the intestinal environment. A growing body of research indicates that probiotics influence host growth, health status, and phenotypic traits by modulating the gut microbiota (30–32). Additionally, probiotics can regulate microbial metabolic activities, thereby linking gut metabolites to host physiological states (33, 34). Guided by this theoretical framework, we analyzed the intestinal microbiota of the nursery pigs. PCoA analysis revealed that Ef1 supplementation significantly altered the gut microbial composition. Through LEfSe analysis and Random Forest analysis at the genus level, we observed a significant increase in the abundance of *Ruminococcus* and *Acetobacter* in the 0.1% HDRsEf1 group. *Acetobacter*, belonging to acetic acid bacteria, can convert ethanol into acetate (35). Previous studies have shown that *Lactobacillus* and *Acetobacter* coexist in the intestinal ecosystem, promoting host health through synergistic metabolic interactions, including the production of short-chain fatty acids (SCFAs) (36, 37). Consistent with these reports, *Lactobacillus* also exhibited higher abundance in the 0.1% HDRsEf1 group in this experiment (*p* > 0.05). Moreover, in ruminants, the abundance of *Acetobacter* is positively correlated with SCFAs such as acetate, propionate, and butyrate (38). Similarly, *Ruminococcus* is a key cellulose-degrading bacterium capable of generating SCFAs through the fermentation of resistant starch from legumes and whole grains. Aligning with our findings, Yang et al. (39) reported that probiotic-fermented additives significantly increased the abundance of SCFA-producing bacteria, including *Ruminococcus*. The abundance of *Ruminococcus* may also mitigate intestinal dysbiosis and potentially help prevent allergies and obesity in infants (40, 41). *Acinetobacter*, a genus of Gram-negative bacteria belonging to the phylum Proteobacteria, is a primary cause of infections in immunocompromised patients in hospital settings (42), with *Acinetobacter baumannii* being particularly notable (43, 44). KEGG pathway predictions showed that all significantly enriched pathways were observed in the 0.1%HDRsEf1 group, encompassing multiple nutrient metabolism and biosynthesis pathways, including Vitamin B6 metabolism. Swine lack the ability to synthesize vitamin B6 endogenously, and Zhu et al. (45) demonstrated that this vitamin mitigates type 2 inflammatory responses by regulating IL-33 homeostasis. Furthermore, studies have shown that inflammation, immune activation, and related diseases are linked to a reduction of up to 50% in plasma levels of active vitamin B6 (46). These findings suggest that Ef1 supplementation reshaped gut microbial functions, favoring pathways involved in inflammation-related and nutrient metabolism processes, which may positively impact host health and ADG.

The 0.1% HDRsEf1 group showed an increase in metabolites such as *trans*-cinnamate, oleic acid, and 7α-hydroxycholesterol. *Trans*-cinnamate, also known as cinnamic acid, has been demonstrated to exert anti-inflammatory effects by modulating the TLR2 and TLR4 signaling pathways (47). Furthermore, cinnamic acid exhibits cardioprotective, anti-inflammatory, lipid-improving, and anti-diabetic properties in diabetic cardiomyopathy (48). Oleic acid, a monounsaturated fatty acid, enhances endurance in mice by inducing PGC-1β expression and altering muscle fiber types (49). It is also recognized as an anti-inflammatory immunomodulator with positive effects on cardiovascular and brain health (50, 51). 7α-Hydroxycholesterol, a key intermediate in bile acid biosynthesis, indirectly modulates intestinal inflammation through bile acid metabolism (52, 53). Conversely, palmitic acid was significantly reduced in the 0.1% HDRsEf1 group. Excessive palmitic acid has been associated with dyslipidemia, hyperglycemia, ectopic fat accumulation, and exacerbated inflammation via TLR4 activation (54), and it is known to promote hepatocyte apoptosis by inhibiting insulin signaling pathways (55). These metabolomic findings are consistent with the ELISA and PICRUSt2 analyses, indicating that Ef1 supplementation enhances the abundance of anti-inflammatory metabolites.

Correlation analysis revealed extensive associations among differential microbial taxa, differential metabolites, and host phenotypes (**Figures 7A–C**). Specifically, *Acetobacter*, which was significantly enriched in the Ef1-supplemented group, exhibited positive correlations with SOD and IL-10 levels, while showing a negative correlation with IL-6 (**Figure 7A**). These metabolic-phenotypic associations were further supported by the positive correlation between *Acetobacter* and its associated anti-inflammatory metabolites (**Figure 7C**). These findings are consistent with previous reports suggesting that certain beneficial taxa may be linked to the modulation of host inflammatory responses (Zhang, 56). In contrast, potentially opportunistic pathogens such as *Acinetobacter* showed negative associations with ADG and were positively correlated with pro-inflammatory markers. Furthermore, the integrated analysis identified a cluster of metabolites that mediated the statistical links between these microbial shifts and host health markers. Collectively, rather than implying direct causality, these results demonstrate that Ef1 supplementation is associated with a coordinated shift in both the gut microbiota and the metabolomic profile, characterized by an increase in “beneficial-associated” taxa (e.g., *Acetobacter*) and a reduction in “risk-associated” taxa (e.g., *Acinetobacter*), which collectively align with the improved growth performance and physiological status observed in nursery piglets.

## Data Availability

The 16S rRNA gene sequencing data have been deposited in the NCBI Sequence Read Archive (SRA) database. The data can be accessed under the accession number PRJNA1417672.

## Supplementary material

Supplementary Table S1 Composition of experimental pig feed (air-dry basis).

Supplementary Table S2 Overview of enriched metabolite sets (Top 20).

Supplementary Figure S1 Microbiome study of experimental pigs.

Supplementary Figure S2 Distribution of annotated metabolite classes and the reliability of the OPLS-DA model was confirmed through permutation testing.
