# Supplementary material for: Enterococcus faecium HDRsEf1 induces changes in gut microbiota and metabolites to maintain host health
Source: Front Immunol. 2026 Apr 15;17:1760841. doi: 10.3389/fimmu.2026.1760841 (PMC13124608; doi:10.3389/fimmu.2026.1760841)
Supplement: Supplementary Table S1 — Composition of experimental pig feed (air-dry basis). [file DataSheet1.pdf]

**Table S1** Composition of Experimental Pig Feed (air-dry basis)

| Items | Content |
|---|---|
| Ingredients | |
| Corn | 60% |
| Wheat | 10% |
| barley | 5% |
| Soybean meal | 18% |
| Fermented soybean meal | 3% |
| Soybean oil | 0.50% |
| Premix | 4% |
| Total | 100% |
| Nutrient levels | |
| Digestible Energy /（MJ/Kg） | 10.04 |
| Crude Protein | 16.50% |
| Digestive lysine | 1.10% |
