# Supplementary material for: Enterococcus faecium HDRsEf1 induces changes in gut microbiota and metabolites to maintain host health
Source: Front Immunol. 2026 Apr 15;17:1760841. doi: 10.3389/fimmu.2026.1760841 (PMC13124608; doi:10.3389/fimmu.2026.1760841)
Supplement: Supplementary Table S2 — Overview of enriched metabolite sets (Top 20). [file DataSheet2.pdf]

Table S2 Overview of Enriched Metabolite Sets (Top 20)

Pathway	level1	level2	DEM	Total	Pvalue	FDR	
Central carbon metabolism in cancer	Human Diseases	Cancer: overview	3	37	0.000313	0.014389	M175T44.pos(L-Arginine|C00062)M180T54.neg(L-Tyrosine|C00082);M339T488.neg(Fructose 1,6-bisphosphate|C00354)
Phenylalanine metabolism	Metabolism	Amino acid metabolism	3	49	0.000722	0.016595	M147T318.neg(trans-Cinnamate|C00423)M180T54.neg(L-Tyrosine|C00082);M165T95.neg(cis-3-(Carboxy-ethyl)-3,5-cyclo-hexadiene-1,2-diol|C11588)
Phenylalanine, tyrosine and tryptophan biosynthesis	Metabolism	Amino acid metabolism	2	35	0.007325	0.102777	-M180T54.neg(L-Tyrosine|C00082);M339T488.neg(Fructose 1,6-bisphosphate|C00354)
Biosynthesis of amino acids	Metabolism	Global and overview maps	3	128	0.011149	0.102777	M175T44.pos(L-Arginine|C00062)M180T54.neg(L-Tyrosine|C00082);M129T153.neg((S)-3-Methyl-2-oxopentanoic acid|C00671)
Protein digestion and absorption	Organismal Systems	Digestive system	2	47	0.012964	0.102777	M175T44.pos(L-Arginine|C00062)M180T54.neg(L-Tyrosine|C00082)
mTOR signaling pathway	Environmental Information Processing	Signal transduction	1	4	0.014948	0.102777	M175T44.pos(L-Arginine|C00062)-
Aminoacyl-tRNA biosynthesis	Genetic Information Processing	Translation	2	52	0.015729	0.102777	M175T44.pos(L-Arginine|C00062)M180T54.neg(L-Tyrosine|C00082)
Fatty acid biosynthesis	Metabolism	Lipid metabolism	2	58	0.019353	0.102777	M283T644.pos(Oleic acid|C00712)M255T651_2.neg(Palmitic acid|C00249)
Melanogenesis	Organismal Systems	Endocrine system	1	6	0.022343	0.102777	-M180T54.neg(L-Tyrosine|C00082)
Chagas disease	Human Diseases	Infectious disease: parasitic	1	6	0.022343	0.102777	M175T44.pos(L-Arginine|C00062)-
Cocaine addiction	Human Diseases	Substance dependence	1	7	0.026021	0.108059	-M180T54.neg(L-Tyrosine|C00082)
Ubiquinone and other terpenoid-quinone biosynthesis	Metabolism	Metabolism of cofactors and vitamins	2	71	0.028281	0.108059	M147T318.neg(trans-Cinnamate|C00423)M180T54.neg(L-Tyrosine|C00082)
Biosynthesis of unsaturated fatty acids	Metabolism	Lipid metabolism	2	74	0.030538	0.108059	M283T644.pos(Oleic acid|C00712)M255T651_2.neg(Palmitic acid|C00249)
Amphetamine addiction	Human Diseases	Substance dependence	1	9	0.033338	0.109539	-M180T54.neg(L-Tyrosine|C00082)
Alcoholism	Human Diseases	Substance dependence	1	10	0.036977	0.113397	-M180T54.neg(L-Tyrosine|C00082)
Prolactin signaling pathway	Organismal Systems	Endocrine system	1	11	0.040604	0.116735	-M180T54.neg(L-Tyrosine|C00082)
Dopaminergic synapse	Organismal Systems	Nervous system	1	12	0.044217	0.119646	-M180T54.neg(L-Tyrosine|C00082)
Amoebiasis	Human Diseases	Infectious disease: parasitic	1	13	0.047818	0.122201	M175T44.pos(L-Arginine|C00062)-
Amyotrophic lateral sclerosis	Human Diseases	Neurodegenerative disease	1	14	0.051406	0.124456	M175T44.pos(L-Arginine|C00062)-
AMPK signaling pathway	Environmental Information Processing	Signal transduction	1	22	0.079657	0.170411	-M339T488.neg(Fructose 1,6-bisphosphate|C00354)
