# Supplementary figures and images for: Enterococcus faecium HDRsEf1 induces changes in gut microbiota and metabolites to maintain host health
Source: Front Immunol. 2026 Apr 15;17:1760841. doi: 10.3389/fimmu.2026.1760841 (PMC13124608; doi:10.3389/fimmu.2026.1760841)

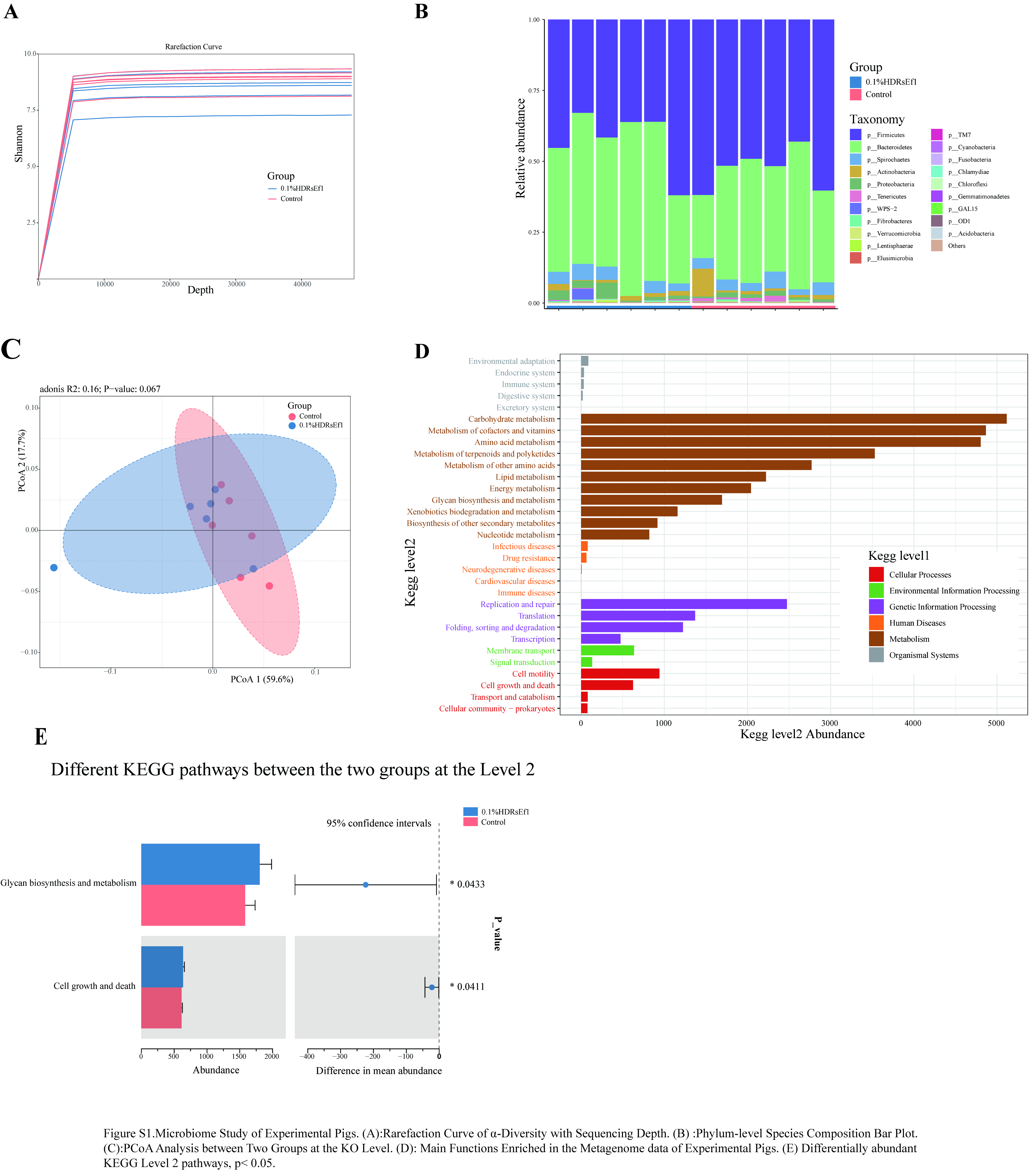

Supplement: Supplementary Figure S1 — Microbiome study of experimental pigs. [file Image1.tif]

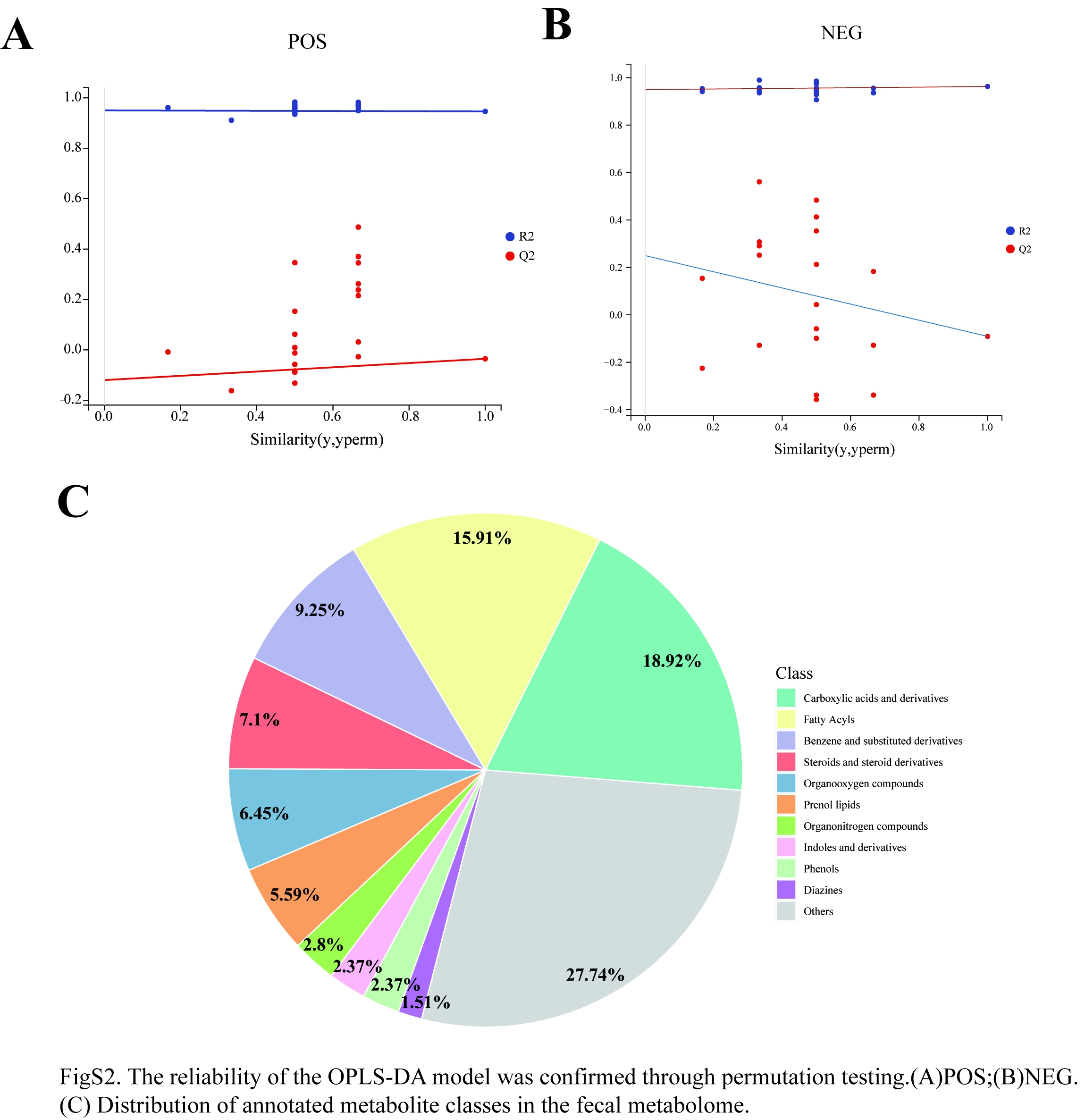

Supplement: Supplementary Figure S2 — Distribution of annotated metabolite classes and the reliability of the OPLS-DA model was confirmed through permutation testing. [file Image2.tif]
